# Benefit–cost analysis of an integrated package of interventions during preconception, pregnancy and early childhood in India

**DOI:** 10.1136/bmjgh-2023-013659

**Published:** 2025-04-12

**Authors:** Tarun Shankar Choudhary, Sarmila Mazumder, Sunita Taneja, Ranadip Chowdhury, Ravi Prakash Upadhyay, Sitanshi Sharma, Neeta Dhabhai, Ole Frithjof Norheim, Nita Bhandari, Kjell Arne Johansson

**Affiliations:** 1 Centre for Intervention Science in Maternal and Child Health, Department of Global Public Health and Primary Care, University of Bergen, Bergen, Norway; 2 Society for Applied Studies, New Delhi, India; 3 Bergen Centre for Ethics and Priority Setting in Health, Department of Global Public Health and Primary Care, University of Bergen, Bergen, Norway

**Keywords:** Health economics, Public Health

## Abstract

**Background:**

We have previously shown that an integrated intervention package delivered during preconception, pregnancy and early childhood substantially reduces low birth weight and stunting at 24 months of age compared with routine care. Now we conduct a benefit–cost analysis to estimate the return on investment of this integrated approach in India. This increases the policy relevance of trial results, given the low investment in healthcare in India.

**Methods:**

We used data from 13 500 participants in the Women and Infant Integrated Interventions for Growth Study (WINGS). Integrated delivery of healthcare, nutrition, water, sanitation and hygiene (WaSH), and psychosocial care interventions during preconception period, or pregnancy and early childhood, or both (full package), was compared with routine care. We converted reduction in mortality, morbidity and increase in productivity to monetary values and calculated the benefit–cost ratio. We used primary and secondary trial health outcomes from the WINGS trial to calculate benefits, and we collected costs prospectively during the trial. Uncertainty was explored in a one-way sensitivity analysis. We applied a discount rate of 3% per annum to both costs and benefits, considering the purchasing power parity (PPP) of US dollars in 2021.

**Results:**

Every dollar invested returned 6.1$ PPP for interventions during preconception, 9.9$ PPP for pregnancy and early childhood interventions and 3.7$ PPP for the full package of interventions compared with routine care in the base case scenario. The return to investment was positive (>4.6$ PPP per 1$ PPP invested) for pregnancy and early childhood interventions in all scenarios of the sensitivity analysis. The net monetary benefits of the interventions ranged between 7364 and 25 917$ PPP.

**Conclusion:**

Our results suggest that integrated and concurrent delivery of healthcare, nutrition, WaSH and psychosocial care interventions during pregnancy and early childhood yield positive economic returns.

**WHAT IS ALREADY KNOWN ON THIS TOPIC**

- Individual maternal and childhood interventions have been shown to yield positive return on investment, that is, a benefit–cost ratio >1 across low-income and middle-income settings.

**WHAT THIS STUDY ADDS**

- This is the first study to estimate the benefit–cost ratio of an integrated package of maternal and child health interventions delivered through preconception, pregnancy and early childhood.
- We show that the return to investment was positive for interventions during pregnancy and early childhood period.

**HOW THIS STUDY MIGHT AFFECT RESEARCH, PRACTICE OR POLICY**

- Implementation of an integrated package of maternal and child health interventions is a good investment and can be considered for scale-up in programme settings.

## Introduction

 The first 1000 days of life from conception to 2 years of age are critical for survival, optimal growth and brain development.^1 2^ The foundations of adult health and social and economic productivity are laid in this period.^3^ Low birth weight infants are at increased risk of adverse outcomes like mortality and morbidity in short term as well as adverse metabolic profile and non-communicable diseases later in life. Stunting in the first 2 years of life is associated with reduced adult height, lower educational attainment and lower cognitive ability.^3 4^ Wasting in early life is associated with higher mortality, morbidity and may also affect long-term neurodevelopment.^2 3 5^ Poor neurodevelopment in early life directly affects social and economic potential in later life. Interventions aimed at reducing the burden of low birth weight, wasting and stunting can thus not only reduce mortality and morbidity but also improve economic productivity and human capital at individual as well as population level.^4 6^

Health benefits from the Women and Infants Integrated Interventions for Growth Study (WINGS), an individually randomised controlled factorial trial conducted in urban and peri-urban low-socioeconomic to mid-socioeconomic neighbourhoods in Delhi India, were substantial.^7 8^ We showed up to 24% reduction in proportion born with low birth weight and up to 49% and 32% reduction in proportion stunted and wasted, respectively, at 24 months of age. Additionally, the intervention led to other benefits like reduction in anaemia and morbidities among women during preconception and pregnancy and improved cognition among children during the first 2 years of life.^8^ However, given the low per capita spending on health in India, evidence on economic returns from an intervention will help policymakers prioritise interventions for scale-up in national programmes.

Benefit–cost analysis (BCA) and other forms of economic evaluations are powerful tools, encouraging the systematic collection and assessment of the evidence needed to support sound and efficient policy decisions.^9^ In low-income and middle-income countries, where resources are scarce, with high competing demands, such decisions are particularly difficult and economic evaluations can be especially helpful. Earlier analyses have shown that maternal and child health interventions have a positive return on investments and compare favourably to investments in sectors like education.^10 11^ BCA aims to assess the effects of policies on overall welfare rather than solely on health. It uses monetary values to measure the extent to which individuals are willing to exchange their income—which can be spent on other things—for the health and non-health outcomes they will likely experience if a policy is implemented. Standard epidemiological studies can only provide evidence of impact on health outcomes. BCA can provide estimates of financial return to investments across different interventions and sectors, thus helping in more effective allocation of funds.

The return on investment of an integrated approach for improving maternal and child health outcomes is unknown. We conducted a BCA by estimating all costs and benefits in monetary terms. Our objective was to estimate the benefit–cost ratio (BCR) of an integrated package of health, nutrition, water, sanitation and hygiene (WaSH), and psychosocial care interventions delivered concurrently either during the preconception period or during pregnancy and early childhood or throughout preconception, pregnancy and early childhood compared with routine care. We measured maternal outcomes, during preconception and pregnancy, and child outcomes at birth and 24 months of age.

## Methods

### Study design, setting and participants

**Figure 1:**
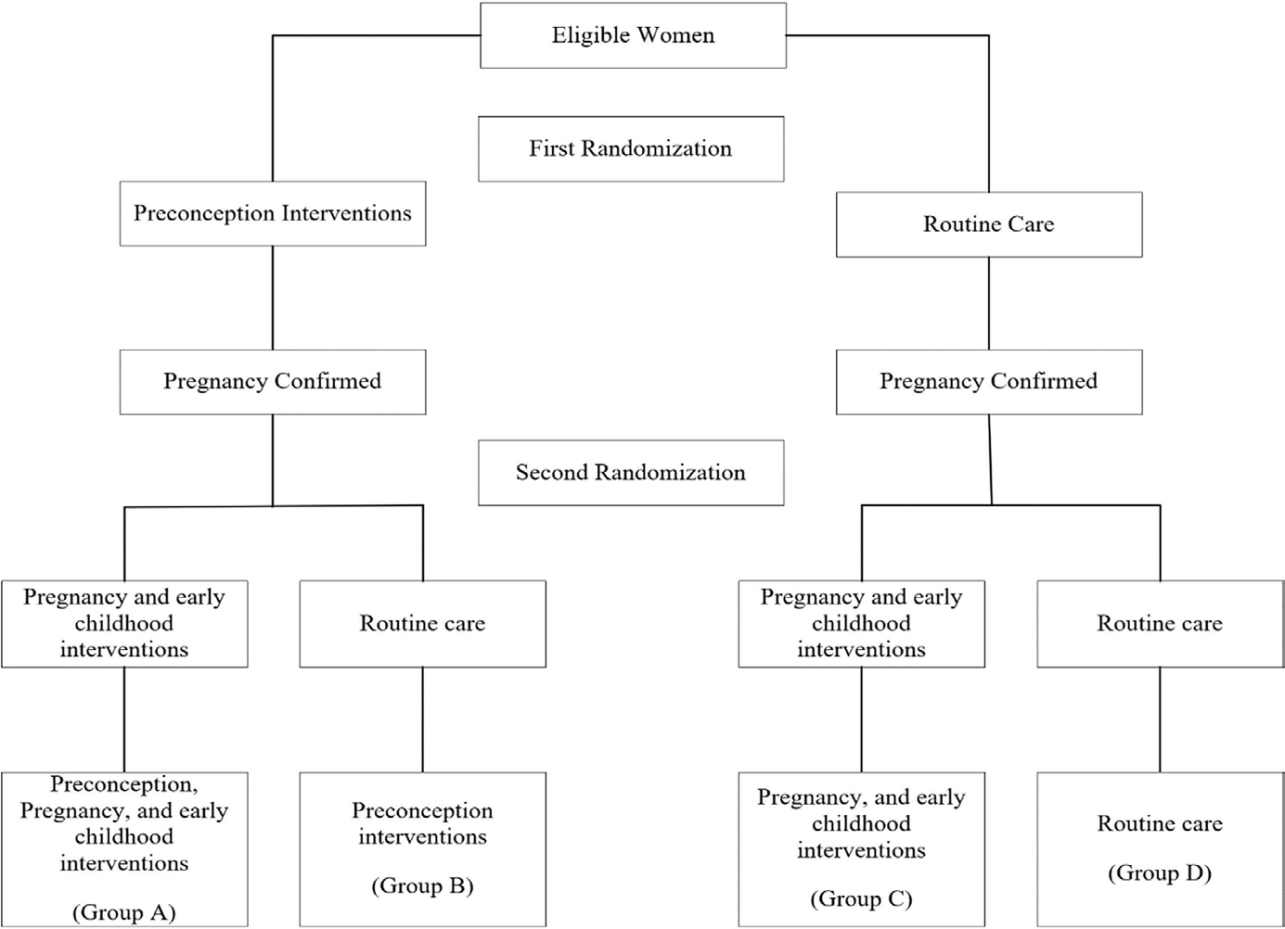
Study design of WINGS.

The BCA was conducted alongside a non-blinded individually randomised controlled trial with factorial design done in urban and peri-urban low to mid-socioeconomic neighbourhoods in South Delhi, India.^8^ Cost data were collected prospectively. The protocol and results from WINGS are reported elsewhere.^7 8^ The trial enrolled and randomised 13 500 married women aged 18–30 years to receive either the preconception intervention package or routine care (first randomisation). Interventions were delivered until women were confirmed to be pregnant or completed 18 months of follow-up after first randomisation. Once pregnancy was confirmed on ultrasound, women were randomised again (second randomisation) to receive either the intervention package for pregnancy and early childhood period or routine care. Newborns were followed up till 24 months of age. The study design has been summarised below (figure 1).

### Intervention

**Figure 2:**
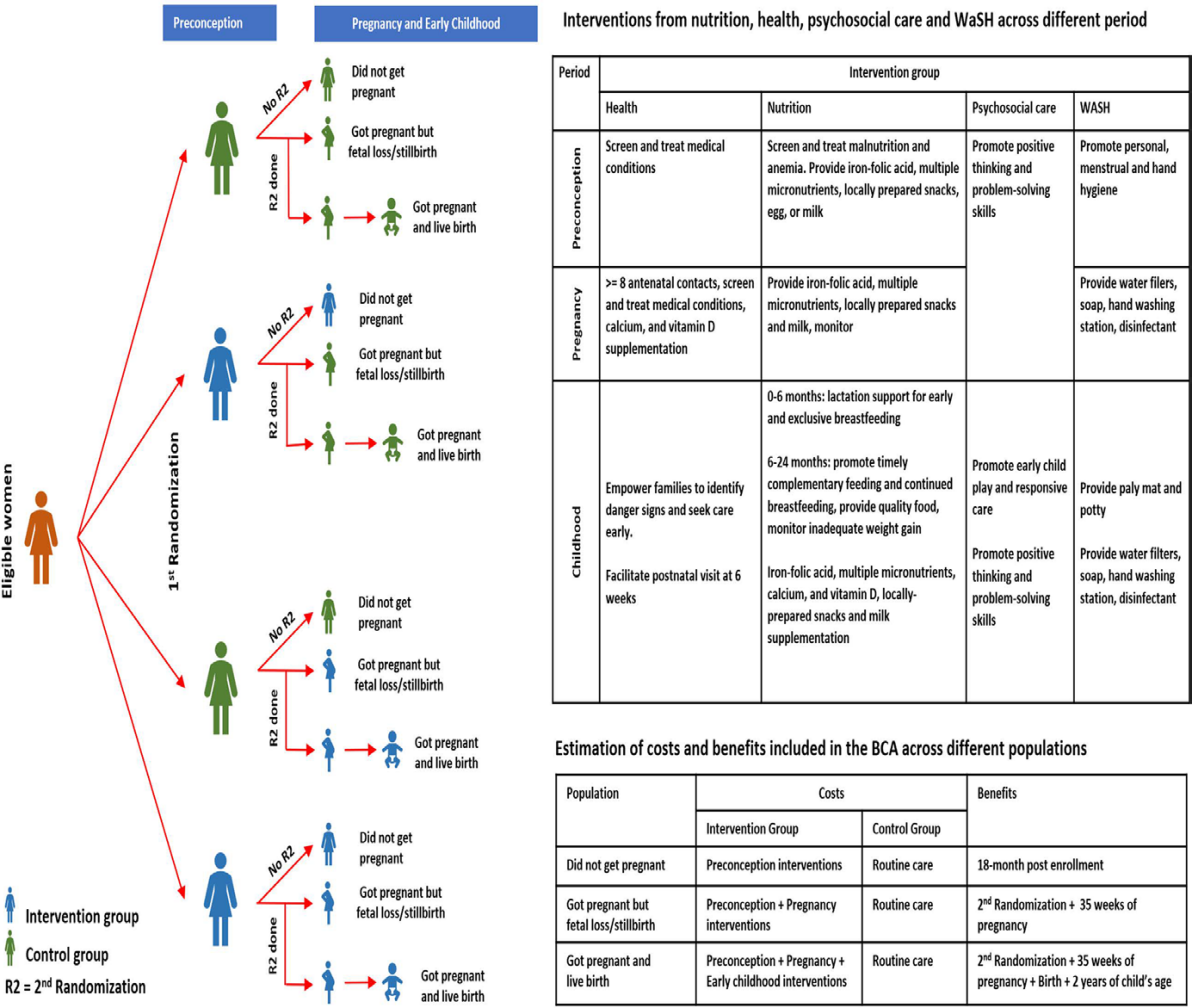
Possible outcomes for a women enrolled across the 4 groups and the costs and benefits for each due to factorial design of the primary trial included in the BCA*.*

The study interventions comprised of evidence-based interventions across the domains of health, nutrition, psychosocial care and WaSH concurrently delivered during preconception, pregnancy and early childhood (0–24 months) period. The interventions were selected based on evidence of impact on intrauterine growth or growth and/or neurodevelopment in the first 2 years of life. Interventions in each of the health service packages are summarised in figure 2. More details on selection of the interventions are available elsewhere.^7 8^ The interventions were delivered at the study clinic and at home through visits by the study team.

The study workers made weekly home visits during preconception. Pregnancy was identified through monthly contacts and confirmed through transabdominal ultrasound. During pregnancy, home visits were made monthly till 32 weeks of gestation, fortnightly between 32 and 36 weeks and weekly thereafter till delivery. The study team visited 6 days a week to directly observe feeding of milk/egg and hot cooked meal. Women in the intervention group were screened for morbidities during preconception and treated according to standard clinical protocols. Women were screened for morbidities and depressive symptoms. Gestational weight gain was monitored during monthly antenatal care visits and treated accordingly. Hospital registration for childbirth was encouraged.

Postdelivery, visits to facilities for postnatal care were encouraged. Postnatal care for mothers and newborns was additionally provided through regular home visits. Newborns were visited at home within 24 hours of birth or hospital discharge, five times during the first month, monthly until 12 months and 3 monthly thereafter up to 24 months of age. Additional visits were made for babies born preterm, low birth weight (LBW) and for mothers with breastfeeding problems. Weights were measured during home visits and children with inadequate weight gain were referred to lactation counsellors and paediatricians. Development milestones were assessed every 3 months by the study team. Compliance with interventions was assessed by study team through observation or by asking mothers during home visits. The control group participants were encouraged to receive care from the existing public healthcare system. Detailed comparison of services available in the existing public healthcare system and those provided to the intervention groups are provided in online supplemental appendix table A1–A3.

### Patient and public involvement

The interventions in the primary trial were informed through formative research conducted for a year to understand the needs and aspirations of the families, to assess their behaviour and perceptions about the interventions in different domains and to determine the best way of delivering interventions at home. We plan to organise dissemination meetings to share the findings with the community and policymakers.

### Outcome assessment

Outcomes were assessed by an independent team at confirmation of pregnancy, at the end of the preconception period, after 26–28 and 35–37 weeks of gestation, within the first week of birth, at 1 month, and 3 monthly thereafter until infant age was 24 months. The newborn’s weight, length, mid-upper arm circumference and head circumference were measured by a pair of workers independently and repeated if the difference was outside the prespecified limit. The two readings were averaged and used for analysis. Ten per cent of measurements were repeated independently. The serious adverse events for this study were severe allergic reactions to supplements and death; these were reported to the Data Safety Monitoring Committee (DSMC) and the ethics committee.

### Study design

The BCA estimated the incremental costs and benefits of the WINGS intervention package compared with routine care where women during preconception, pregnancy and early childhood continued to receive care from the public healthcare system (figure 2). We modelled the costs and benefits over the lifetime for the study participants using the provider (trial) perspective as all the services as part of the intervention package were delivered by the study team. Data from WINGS were used for estimating the input parameters for the model. We followed BCA reference case guidelines and adhered to the Consolidated Health Economic Evaluation Reporting Standards.^9 12^ All analyses are reported in 2021 International $ purchasing power parity (PPP) with a conversion rate of 1$ PPP=23.13 Indian Rupee.

### Benefit estimation

Outcomes assessed during preconception and pregnancy among women and at birth and 2 years of age among their children were used for benefit estimation (online supplemental appendix table A4). We used the data on efficacy of the intervention(s) from the primary trial and converted them to monetary values. Total monetary benefit was calculated as benefit from reduced mortality (for stillbirths and mortality in the first 2 years of life), reduced morbidity (for women during preconception and pregnancy) and increased productivity (for low birth weight, nutritional and neurodevelopmental outcomes at 24 months in children). Present value of total benefits was estimated by adding the benefits to women and children using the following equations.


$$Total\;Benefit_{\;Present}=Total\;Benefit_{\;Women}+Total\;Benefit_{\;Children}$$

$Total\;Benefit\;Women=\sum_{t=0}^{T1}P_{Women}*B_{Women}{\left(1+r\right)}^{-t}$

$Total\;Benefit_{\;Children}=\;\sum_{t=0}^{T2}P_{Children}*B_{Children}{\left(1+r\right)}^{-t}$

where Total Benefit _Present_ = present value of total benefits to all women and children in the study population; B_Women_=value of the benefits to each woman in the preconception and pregnancy period; B_Children_=value of the benefits to each child during their lifetime; r=discount rate; P_Women_=number of woman in the intervention groups; P_Children_=number of children in the intervention groups; T1=time duration for which the benefits persist among women; T2=expected life expectancy at the age of 2 years.

We used the value of statistical life (VSL) approach as recommended in the BCA reference case to estimate the value of mortality reduction.^13^ VSL describes the willingness of those affected by a policy to exchange their own income for the risk reductions they experience. We calculated the VSL estimates for India based on the gross national income (GNI) per capita PPP:VSL ratio of 100 with an income elasticity of 1, without any age adjustment.^13 14^ The impact on morbidity/illness reduction was calculated using the VSL year (VSLY) approach.^13^ We estimated years lost to disability at a constant VSLY. We calculated VSLY as VSL/35, as 35 years has been commonly used as duration of productive life in the literature.^13^ We calculated the change in morbidity by subtracting the years lived with disability between the intervention and control arms and multiplying it with the disability weight as provided in Global Burden of Disease (GBD) estimate 2019. One disability adjusted life year (DALY) was valued at one VSLY.^11^ Outcomes which were measured but did not have disability weights available in the GBD 2019 estimates were excluded from the analyses.^15^ Earlier work by Alderman *et al* and Galasso *et al* shows that the gain in productivity is an indirect result from one or more of the following: improved cognition, improved height and higher educational attainment. We estimated the expected lifetime productivity benefits by summing up the benefits from higher birth weight, improved cognition, improved height, reduced malnutrition and higher educational attainment, as applicable for each of the outcomes of interest.^6 10 16^ Since the pathways through which they lead to improved productivity overlap, adjusted estimates from literature were used.^16^

### Measurement of resource use and costs

#### Costing the integrated intervention package

The unit cost for delivering different components of the intervention package across the domains of health, nutrition, psychosocial care and WaSH was calculated using a top-down ingredient-based approach. The participants in the intervention arm had access to all the services available in the existing public healthcare system similar to the control arm participants and the associated costs were also the same. The cost of the interventions was borne by the trial, and the participant did not pay for the intervention package out-of-pocket. Hence, we calculated the incremental costs of the integrated intervention package in the intervention arms of the study. The total cost of delivering each of the intervention packages was generated for Preconception, Pregnancy and Early Childhood Care; Preconception only, and Pregnancy and Early Childhood Care only by multiplying the unit cost for the intervention package by the number of participants, to whom it was provided.

The incremental cost was mainly due to additional interventions in addition to services currently available in the routine healthcare system. Delivery of all components of the intervention with high compliance was ensured by dedicated teams, which was a major driver of cost.

The protocol mandated costs such as administrative, accounting, standardisation and measurements of outcomes. Costs for activities that were not directly related to the delivery of intervention or did not impact the outcomes were not included.^17^ Shared costs for items partially used for delivery of interventions or choosing which interventions to deliver, were apportioned based on the appropriate proportion of utilisation (eg, proportion of time used). Costs for components that were used/shared across the study periods (Preconception, Pregnancy and Early Childhood Care) and/or the study groups were apportioned to each period.

Costs were estimated for human resources, equipment, training, consumables and transportation. We used standard economic costing methods to cost for equipment and the life of the equipment was taken into consideration. Costs for donated items such as water filters anthropometry equipment used by intervention development team were also included. The costs of quality control activities carried out by the intervention delivery teams, including diagnostic investigations such as ultrasound to determine intrauterine growth restriction—which informed the provision of specific intervention components—were included in the costing

Since participants in the intervention arm across different phases of the study had access to care from the public healthcare system, similar to the control group, the incremental costs were calculated as:

Incremental cost of interventions=(cost of interventions+cost of routine care)−cost of routine care

Our assumption would lead to higher incremental cost and lower BCR, in case the utilisation of services from the existing public healthcare system was lower.

Incremental unit cost of the intervention for the three groups was generated as shown below:

**Table:** 

Incremental unit cost of intervention for preconception (group B)	$=UC_{\;Preconception}*\;(N_{\;Preconception\;Group}\;/\;N_{\;Children\;24m})$
Incremental unit cost of intervention for pregnancy and early childhood (group C)	$=UC_{\;Preconception}*\;(N_{\;Preconception\;Group}\;/\;N_{\;Children\;24m})\;+\;\;UC_{\;Pregnancy}*(N_{\;Pregnancy}\;/\;N_{\;Children\;24m})\;+\;UC_{\;Early\;Childhood}$
Incremental unit cost of intervention for preconception, pregnancy and early childhood (group A)	$=UC_{\;Preconception}\;*\;(N_{\;Preconception\;Group}\;/\;N_{\;Children\;24m})\;+\;\;UC_{\;Pregnancy}\;*\;(N_{\;Pregnancy}\;/\;N_{\;Children\;24m})\;+\;UC_{\;Early\;Childhood}$

UC = Unit Cost

#### BCR and net monetary benefit

We calculated the BCR by dividing the incremental benefits in monetary terms between the three intervention arms (Preconception, Pregnancy and Early Childhood Care, Preconception only, and Pregnancy and Early Childhood Care only) and the control arm (routine care) by the incremental cost of the intervention package. Future savings on healthcare costs due to reduced morbidity and improved growth and neurodevelopment were counted as benefits for calculating BCR. A BCR greater than 1.0 indicates that benefits outweigh costs, that is, a positive net present value. A BCR less than 1.0 indicates that the costs outweigh the benefits leading to a negative net present value. Net monetary benefit (NMB) was calculated by subtracting the incremental costs from the incremental benefits in monetary terms.^18^

#### Discounting

Different discounting rates, that is, 3%, 5% and 7%, were used for both costs and health benefits.^17 18^ The duration of discounting was different for the inputs (costs) and the outputs (health benefits) as the time duration of these did not overlap. The costs for different packages, that is, preconception, pregnancy and early childhood were incurred for varying duration, during the trial period of 4 years. The benefits, however, would occur over the lifetime for children or persist for few months to years, after stopping interventions among women.

#### Missing data

As shown in figure 2, the number of participants for whom outcomes and costs were assessed varied across different phases of the study.^8^ Only a subset of participants to whom preconception interventions were delivered got pregnant. Pregnancy loss occurred among participants to whom interventions were delivered during pregnancy period. Additionally, the trial was stopped before all children could complete 24-month follow-up by the DSMC.^8^ We assumed that the costs and outcomes for participants among whom these could not be measured were similar to those where data on costs and outcomes were available. Some children died during early childhood and the average age at mortality was used to estimate the cost of delivering the interventions for these children.

#### Sensitivity analysis

We carried out one-way sensitivity analyses to explore uncertainty surrounding estimates. Sensitivity analyses were conducted for the following parameters: effect size for all outcomes for which benefits were calculated, discount rate, rate of economic growth, VSL, VSLY and cost of delivering the interventions. The base, low-case and best-case values for the parameters are available in online supplemental appendix table A5. We present the sensitivity analysis using tornado plots.

#### Role of funding source

The study was funded by Biotechnology Industry Research Assistance Council of the Department of Biotechnology, Government of India (GCI-ACT ref No BIRAC/GCI/0085/03/14-ACT) and Bill and Melinda Gates Foundation, USA (grant ID OPP1191052). The funders had no role in study design, data collection, analyses, interpretation of data, writing of the report or decision to submit the article for publication. The corresponding author had full access to all the data in the study and takes responsibility for the integrity of the data and the accuracy of the data analysis.

## Results

The intervention was delivered to 6722 women in the preconception period, 2460 women during pregnancy and 2344 children during childhood period. The costs and outcomes were both calculated for these numbers. Table 1 presents the effect size of the outcomes assessed in preconception, pregnancy and childhood period included in estimating the benefits for the BCA. The benefits for childhood outcomes were highest in the group which received intervention throughout, that is, during preconception, pregnancy and early childhood period.

**Table 1:** Effect size for outcomes among women and children from WINGS included for estimation of monetary benefits in the analysis

Period	Outcome	Preconception vs routine care	Pregnancy and early childhood vs routine care	Preconception, pregnancy and early childhood vs routine care
		ARR (%) or MD (98.3% CI)	ARR (%) or MD (98.3% CI)	ARR (%) or MD (98.3% CI)
Preconception	RTI/STI	−6.25(−2 to −10.49)	2.86(7.73 to −2.01)	−5.7(−1.38 to −10.03)
	Mild anaemia	2.34(8.02 to −3.34)	1.11(6.95 to −4.73)	2.3(8.01 to −3.42)
	Moderate anaemia	−14.07(−9.35 to −18.79)	1.34(6.92 to −4.23)	−14.97(−10.22 to −19.73)
	Severe anaemia	−1.91(−0.83 to −2.99)	0.31(1.85 to −1.22)	−1.83(−0.73 to −2.93)
	Depression	0.61(2.15 to −0.93)	0.79(2.36 to −0.78)	−0.15(1.37 to −1.66)
	Hypothyroidism	−0.12(3.17 to −3.41)	2.72(6.34 to −0.89)	−1.24(2.06 to −4.49)
Pregnancy	RTI/STI	−1.67(4.02 to −7.35)	−8.71(−3.34 to −14.07)	−9.76(−4.61 to −14.91)
	Preeclampsia/eclampsia	−0.84(1.33 to −3.02)	−1.76(0.46 to −3.98)	−2.78(−0.66 to −4.9)
	Postpartum haemorrhage	−0.12 (1.83 to −2.07)	0.45 (2.53 to −1.64)	−1.55(0.05 to −3.14)
	Mild anaemia	2.99(8.38 to −2.41)	−4.43(0.51 to −9.38)	−3.67(1.15 to −8.49)
	Moderate anaemia	−3.69(1.56 to −8.93)	−16.57(−12.09 to −21.05)	−14.9(−10.43 to −19.37)
	Severe anaemia	−1.45(0.05 to −2.94)	−2.81(−1.33 to −4.28)	0(0 to 0)
Childhood	Low birth weight	−5.7(−1.12 to −10.28)	−3.4(1.54 to −8.34)	−5.59(−0.85 to −10.32)
	Mortality	−0.96(1.09 to −3.09)	−3.33(−1.16 to −5.51)	−1.24(0.81 to −3.28)
	Stunting at 24 months	0.45(7.15 to −6.26)	−7.54(−1.33 to −13.74)	−7.98(−1.71 to −14.24)
	Wasting at 24 months	2.1(8.5 to −4.31)	−3.82(2.67 to −10.31)	−3.71(2.15 to −9.57)
	Cognition at 24 months	0.09(0.25 to −0.07)	0.16(0.32 to 0)	0.29(0.44 to 0.13)

Absolute risk reduction, adjusted for place of birth, family possesses below poverty line card, woman’s height, woman’s body mass index for potential confounder and twins for clustering within the household. Mortality includes stillbirth and death in the first 2 years of life.

ARR, absolute risk reduction; BSID III, Bayley Scales of Infant and Toddler development, third edition; MD, mean difference; RTI/STI, reproductive tract infection/sexually transmitted infections.

The unit cost of delivering the intervention package by components and across the phases of the study is presented in table 2. The overall unit cost was highest for early childhood phase followed by pregnancy and preconception phase, respectively. Human resources (30% to 40%), nutrition (15% to 46%), transportation (13% to 19%) and health interventions (0.4% to 22%) were the major drivers of total cost across all phases of the study. The average duration of the intervention was 11 months during preconception, 7 months during pregnancy and 24 months for early childhood period and hence the unit cost/month for the pregnancy interventions was highest.

**Table 2:** Unit cost for delivering different components of the intervention during different phases of the study in $ PPP

Component	Preconceptioncost (% of total)	Pregnancycost (% of total)	Early childhood^*^cost (% of total)
Consumables	11.9 (2.4)	16.9 (1.7)	19.8 (1.1)
Equipment	9.1 (1.8)	11.2 (1.1)	27.7 (1.5)
Health	110.4 (22)	183.2 (18.3)	7.3 (0.4)
Human resource	200.7 (39.9)	391.4 (39.1)	564.4 (30.1)
Nutrition	74.9 (14.9)	209.7 (21)	863.6 (46)
Psychosocial care	1.7 (0.3)	1.1 (0.1)	3.5 (0.2)
Transport	93.9 (18.7)	157.2 (15.7)	247.9 (13.2)
WaSH	0 (0)	29.4 (2.9)	143.1 (7.6)
Total	502.5 (100)	1000 (100)	1877.3 (100)
Average duration (months)	11	7	24
Average cost/month	45.7	142.9	78.2

* 0–24 months for child, 0–6 months post-partum for mother.

PPP, purchasing power parity; WaSH, water, sanitation and hygiene.

Table 3 presents the value of benefit for different outcomes, total cost, BCR and NMB across the three comparisons. We calculated the incremental cost of delivering the interventions over and above the cost of utilising the public health system in the intervention and the control arm. The unit of analysis for both benefits and costs was in $ PPP per mother–infant dyad. The unit cost was highest for the group which received preconception, pregnancy and early childhood interventions followed by pregnancy and early childhood and preconception group, respectively.

**Table 3:** Incremental monetary benefits, costs and benefit**–**cost ratio and net monetary benefit of the integrated intervention package for different comparisons per mother–child dyad in $ PPP

Period	Outcome	Preconception vs routine care	Pregnancy and early childhood vs routine care	Preconception, pregnancy and early childhood vs routine care
Preconception	RTI/STI	64.9	−29.7	59.2
	Anaemia	307.7	−36.8	318.4
	Depression	−98.4	−127.5	24.2
	Hypothyroidism	0.9	−21.1	9.6
Pregnancy	RTI/STI	17.4	90.5	101.4
	Preeclampsia/eclampsia	59.5	124.8	197.1
	Postpartum haemorrhage	2.8	−10.5	36.0
	Anaemia	121.0	396.6	241.2
Childhood	Low birth weight	1138.6	679.2	1116.7
	Mortality (stillbirth and death in first 2 years of life)	6844.8	23 742.9	8841.2
	Stunting at 24 months	−130.9	2193.0	2321.0
	Wasting at 24 months	−276.5	3502.9	488.4
	Cognition at 24 months	753.4	1339.4	2427.6
	Benefits	8805.3	28 843.9	16 182.0
	Costs	1441.1	2926.8	4367.9
	Benefit–cost ratio	6.1	9.9	3.7
	Net monetary benefit	7364.2	25 917.1	11 814.1

PPP, purchasing power parity; RTI/STI, reproductive tract infection/sexually transmitted infections.

The monetary value of benefits in table 3 does not add up (preconception+pregnancy, and early childhood period≠preconception, pregnancy and early childhood period) as the impact of interventions was different across the three groups (table 1). However, costs add up since the ratio of women–infant dyad was similar in all groups.

The point estimates for monetary benefits were negative for some outcomes in the intervention groups compared with routine care, but the 95% CIs are not significant (table 1). Childhood outcomes, that is, impact on mortality, low birth weight, stunting, wasting and improved cognitive development were the major contributors to monetary benefits from the intervention. Anaemia was the major driver of monetary benefit in women during preconception and pregnancy. The base estimate of BCR was greater than 1 for all comparisons suggesting that the net present value of the benefits from the intervention was greater than the costs incurred, that is, a positive net present value. Pregnancy and early childhood interventions had a BCR of 9.9 and NMB of 25 917 $ PPP. The BCR (NMB) for preconception package and full package was 6.1 (7364 $ PPP) and 3.7 (11 814 $ PPP), respectively.

**Figure 3:**
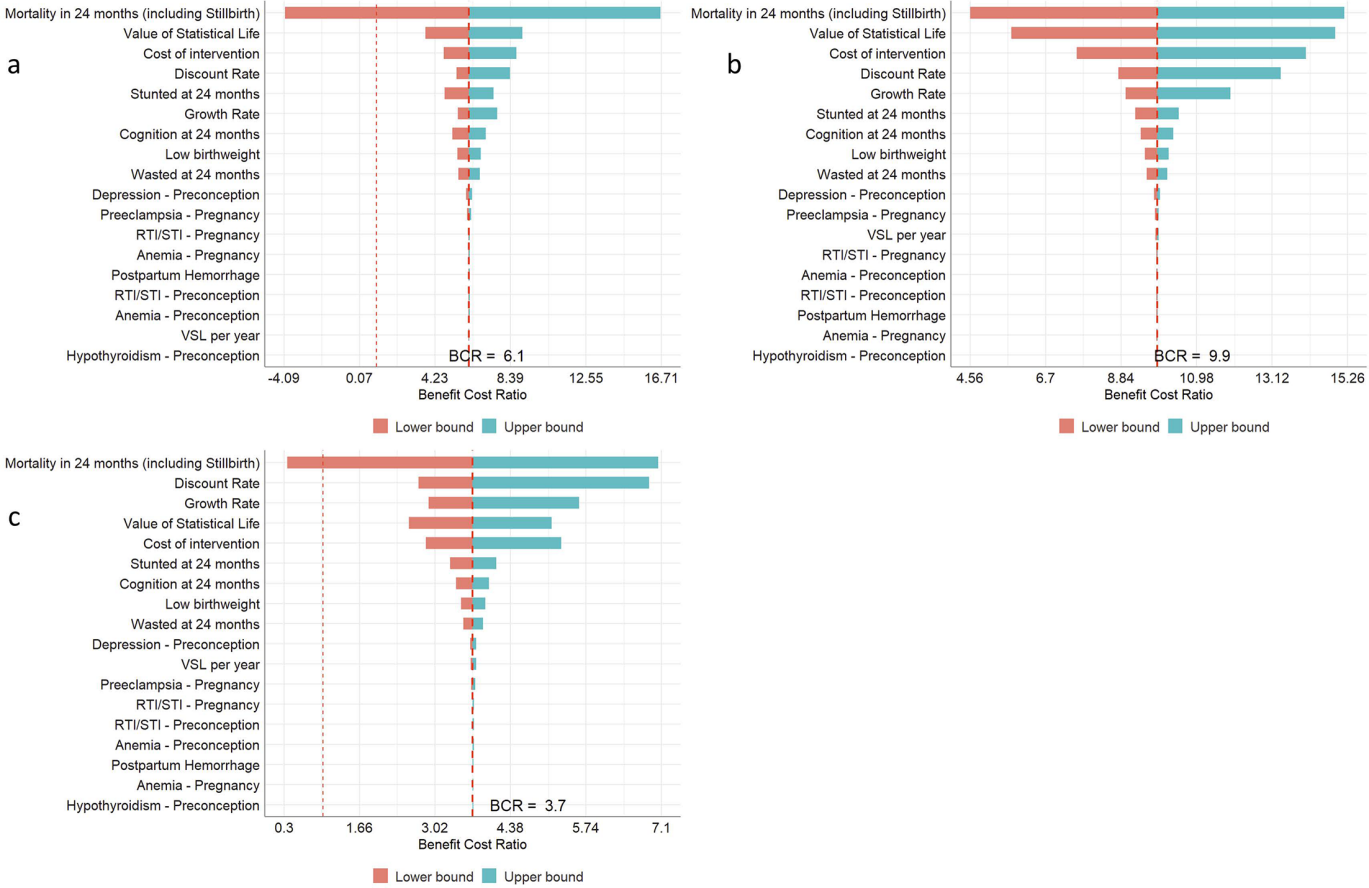
One way sensitivity analysis for different input parameters across the comparison between a) Preconception vs Routine care, b) Pregnancy and Early Childhood Vs Routine care, and c) Preconception, Pregnancy and Early Childhood vs Routine care*.*

Findings from the one-way sensitivity analysis are presented in figure 3. As shown, the low-case and best-case estimate for BCR across different levels of input parameters was greater than 1 for the Pregnancy and early childhood versus routine care. The BCR for the other two comparisons were less than 1 in the low-case scenario, that is, the net present benefit would be less than the cost of the intervention package.

Mortality impact was the primary driver of uncertainty, which led the BCR to be <1 in the low-case scenario. Other major drivers included value of VSL, cost of the intervention page and discount rate used.

## Discussion

Every dollar invested in delivering the integrated intervention package (WINGS) to mother and child returned between 3.7 and 9.9 dollars. The substantial health benefits such as 13%–24% reduction in low birth weight, and 4%–51% reduction in stunting at 24-month among others, had incremental benefits between 7364$ PPP and 25 917$ PPP for the population receiving the intervention. The unit cost of delivering the integrated package of interventions ranged between 1441$ PPP per mother in the preconception group and 4368$ PPP per mother and child in the combined package with preconception, pregnancy and childhood interventions. The return to investment remained positive for all parameters in the sensitivity analysis of pregnancy and early childhood interventions. Mortality (including stillbirth) had the biggest impact on BCR in the sensitivity analysis.

This is the first BCA of an integrated package of interventions like WINGS. As a comparison, the ‘Investment Framework for Nutrition’ by Shekar *et al* and the 2020 ‘WHO–UNICEF–Lancet Commissions’ by Clark *et al* show that different nutritional interventions have the potential to yield positive return to investment.^10 19 20^ The BCR for different maternal and child health interventions ranged between 6 and 15 in their analyses.^10^ BCA for interventions included in the WINGS package like multiple micronutrient and calcium supplementation in pregnancy have been shown to have a BCR of 18.^21^ Breastfeeding promotion (BCR of 5.2 and 24) and complementary feeding promotion (BCR of 8.2 and 36) have been shown to yield economic benefits across different settings.^21 22^ Stenberg *et al* have reported similar BCR of 8.7 for a package of Reproductive, Maternal, Neonatal and Child health (RMNCH) interventions modelled on estimates of impact for interventions from multiple studies across different settings and point of delivery.^20^ Although our methods of estimating monetary value of health benefits were similar, the costs were estimated for scale-up in programme settings. Modelling-based analysis for selected nutritional interventions in pregnancy and early childhood for Haiti showed a BCR of 5.2, although different study design and interventions included preclude a direct comparison of the findings.^11^ The unit costs of scaling up interventions were calculated based on cost of scaling up existing programmes and were therefore much lower compared with our estimates from the trial.

Our estimates for the base case scenario indicate a positive return on investment for the integrated WINGS intervention package under both assumptions, that is, including or excluding the benefit from reduced mortality in the first 2 years of life (online supplemental appendix table A6). Additionally, the BCR was positive for both comparisons as per the factorial design of WINGS (online supplemental appendix table A7). Negative return on investment in the low-case scenario was driven by uncertainty from the mortality estimates in the one-way sensitivity analysis and the fact that only one in three participants who received the interventions became pregnant and had a child for whom outcomes could be estimated during pregnancy and at 24 months of child’s age. The VSL values used in the sensitivity analyses capture the values for VSL reported from Indian settings and hence using Indian VSL estimates will not impact our results.^23 24^ Additionally, the unit cost of the intervention package was high as it was delivered in research settings and human resources and transportation comprised a large proportion of the overall costs. Scaling up the WINGS package in programme settings would be associated with lower costs as most of the components from the pregnancy and early childhood package are already part of the existing national programmes in India. Hence, the incremental cost of improving the coverage and quality of these will be low.

We show that the integrated pregnancy and childhood interventions have a lot of economic value and make a good investment case in similar settings.^8^ Our estimates can be easily modified to estimate the cost of scale-up in the existing programmes elsewhere. For example, the current Integrated Child Development Services scheme has a budget of 10.3$ PPP/month under supplementary nutrition programme for pregnant and lactating mothers compared with 30$ PPP/month in our study under research settings. Centralised procurement, at scale, has been shown to reduce costs for pharmaceutical drugs.^25^ Modelling-based estimates have shown lower costs for intervention delivery in programme settings compared with our research setting estimates.^3 10 11^ Additionally, our results show that the return on investment is positive even in the low-case scenario for pregnancy and early childhood interventions.

Traditionally, economic evaluations of health interventions report findings in terms of Incremental Cost Effectiveness Ratios, which estimate the additional cost per disease-specific outcome or the utility measures DALY or QALY gained. BCA, on the other hand, presents these benefits in monetary terms, which facilitates comparison of results across sectors. Our estimate for the WINGS intervention package compares favourably to, for example, the BCR of 4.3 for investments in clean drinking water and sanitation in the Clean India Mission (Swachh Bharat Mission).^26^ Policymakers can use BCA information to make informed decisions about inclusion of health as well as non-health interventions in national programmes and policies, as decisions to invest in health are seldom within the domain of the health ministry alone.

The current analysis used the cost and effect estimates from a rigorously conducted randomised trial. We included all the benefits for mothers as well as children. Health and productivity benefits were converted to monetary values based on the guidance available in the BCA reference case. Adjusted estimates and 98.3% CIs for effect size were used in the sensitivity analysis, which was conducted for all model parameters. This is the first trial-based BCA for such an extensive and integrated package of interventions. Our findings should be interpreted keeping the following in mind: we did not collect data on resource utilisation from the routine healthcare system in the study. Since there were multiple outcomes with overlapping pathways, some estimates might have been double-counted although we tried to avoid this by adjusting the benefit estimation (using a weighting factor of 0.6). Certain outcomes could not be included in estimating benefits as disability weights were not available for them. Additionally, it was not possible to identify the optimal mix of interventions which yield the highest BCR and NMB as the outcomes as well as costs were assessed for the entire package of interventions and not for individual components.

## Conclusion

Our findings show that integrated and concurrent delivery healthcare, nutrition, WaSH and psychosocial care interventions, during preconception or pregnancy and early childhood or both periods, yield positive economic returns in the base case scenario. The return on investment was positive even in the low-case scenario for pregnancy and early childhood interventions.

## Supplementary material

10.1136/bmjgh-2023-013659 online supplemental material 1

## Data Availability

Data are available upon reasonable request.
