# Supplementary material for: Benefit–cost analysis of an integrated package of interventions during preconception, pregnancy and early childhood in India
Source: BMJ Glob Health. 2025 Apr 12;10(4):e013659. doi: 10.1136/bmjgh-2023-013659 (PMC11997828; doi:10.1136/bmjgh-2023-013659)
Supplement: online supplemental material 1 [file bmjgh-10-4-s001.pdf]

Appendix

Table A1. Comparison of Preconception interventions between the intervention and routine care group

Domain	Intervention Group	Control Group (Routine Care)
Health	Screen and treat medical conditions known to affect fetal and infant growth i.e.,<br>Reproductive tract infections,<br>Syndromic approach (RTI),<br>Symptoms of tuberculosis (TB),<br>Hypertension,<br>Diabetes (HbA1c ≥6.5%) and pre-diabetes (HbA1c 5.7%-6.4%),<br>Hypothyroidism (thyroid-stimulating hormone (TSH) >5.5 mIU/ml or TSH 4.01 to 5.5mIU/l and anti-TPO Ab positive),<br>Hyperthyroidism (<0.4 mIU/ml)<br><br>Provision of contraception to women living with husband for <1 year, having a child aged <1-year, severe malnutrition, moderate to severe anemia, hypothyroidism, RTI/STI and diabetes, hypertension<br><br>Bi-annual deworming (Albendazole 400 mg)	Family planning services including promotion of spacing methods
Nutrition	Weekly iron-folic acid (IFA) supplementation (300 mg ferrous fumarate, 1.5 mg folic acid, 15 mcg cyanocobalamin)<br><br>Multiple micronutrients thrice weekly<br><br>Screening for malnutrition and anemia<br><br>Management of undernutrition<br>BMI <16 kg/m2: Refer to hospital, locally prepared snacks and egg or milk (1000 kcal/day and 20-22 g protein/day)<br>BMI 16 to 18.49 kg/m2: Snacks and egg or milk (500 kcal/day and 12-14 g protein/day)<br>BMI <18.5 to 20.99 kg/m2: Egg or milk (70 kcal, 6 g protein) 6 days a week<br><br>Monthly weight assessment for those with BMI <18.5 kg/m2<br>Nutrition counselling and management of infections for those with inadequate weight gain (IWG, weight gain <500 g per month)<br><br>Anemia<br>Severe anemia (hemoglobin <8 g/dL): Refer to hospital for treatment.<br>Mild to moderate anemia (hemoglobin 8 to 11.99 g/dL): treatment with IFA (300 mg ferrous fumerate, 1.5 mg folic acid, 15 mcg cyanocobalamin)	Weekly IFA supplementation as part of the National Iron plus Initiative Program
WaSH	Promotion of personal, menstrual and hand hygiene	None
Psychosocial support	Screen for depressive symptoms (using PHQ-9).<br>Counselling by study psychologist if PHQ score ≥ 10.<br>Referral to psychiatrist if PHQ-9 score ≥ 15 and/or presence of suicidal ideation.<br><br>Promotion of positive thinking and problem-solving skills<br><br>Screen for substance abuse and exposure to second-hand smoke and alcohol use in husbands and counsel	None

Table A2. Comparison of Pregnancy and Early Childhood interventions between intervention and routine care group

	Intervention Group	Control Group (Routine Care)
Health	At least eight antenatal care (ANC) contacts according to WHO ANC guidelines, registration for institutional delivery<br><br>In addition to hospital-based ANC clinics, increase coverage through free-of-cost, high-quality, study outpatient clinic services within community including laboratory services.<br><br>Screening and treatment for medical conditions: HIV, VDRL, syndromic RTI, syndromic TB, Hepatitis B (HbsAg), hypo- and hyperthyroidism (TSH) assessment at first contact<br><br>Urine routine and microscopic examination and asymptomatic bacteriuria by urine culture four times<br><br>Gestational diabetes by oral glucose tolerance test thrice<br><br>Pregnancy induced hypertension (blood pressure) at every visit.<br><br>Anemia (hemoglobin) four times.<br><br>Tetanus toxoid immunization<br><br>Calcium (1000 mg) and vitamin D (400 IU) supplementation daily starting from second trimester throughout pregnancy.<br><br>Anti-helminthics (Albendazole 400 mg) at 20 weeks	Routine antenatal care which included 4 visits<br><br>Urine testing for sugar and albumin<br><br>Most of the tests were under desirable category as per the national guidelines during the study period (pre-2021) although these were done in the control group as they primarily seek care at a large tertiary care hospital in the public healthcare system.<br><br>Tetanus toxoid immunization<br><br>Calcium (1000 mg) and vitamin D (400 IU) supplementation daily starting from second trimester throughout pregnancy.<br><br>Anti-helminthics (Albendazole 400 mg) at 20 weeks
Nutrition	Counselling<br><br>IFA (100 mg iron, 500 µg folic acid) supplementation daily throughout pregnancy from second trimester<br><br>Multiple micronutrients daily throughout pregnancy Food Supplements<br><br>BMI <25 kg/m2<br>Second trimester: Food supplements (210 kcal, 2 g protein) in the form of a choice of snacks prepared locally and milk (70 Kcal, 6 g protein)<br>Third trimester: Food supplements (400 kcal, 21 g protein) in the form of a choice of snacks prepared locally and milk (70 Kcal, 6 g protein)<br>Extra snacks (500 kcal, 20 g protein) throughout pregnancy, to women with BMI <18.5 kg/m2<br>BMI 25 to <30 kg/m2<br>Milk (70 kcal, 6 g protein) 6 days a week<br>No food supplements to overweight and obese women (BMI > 25 kg/m2)<br>Anemia<br>Severe anemia (hemoglobin <7 g/dL): Refer to hospital for treatment.<br>Mild to moderate anemia (hemoglobin 7 to 10.99 g/dL): treatment with IFA (100 mg iron, 500 µg folic acid) twice daily<br>Gestational weight gain<br>Weight monitoring every month.<br>identification of inadequate weight gain (IWG) according to Institute of Medicine's guidelines<br>Management of inadequate weight gain (IWG):<br>Nutritional Counselling<br>Hot meal (500 Kcal, 20 g protein) 6 days a week till delivery<br>Screening and treatment of infections (UTI, RTI, TB)	Food is provided through Anganwadis although compliance in the control group was not ensured and the uptake in general was low based on national level surveys.<br><br>Supplementary food for mothers, either cooked food or take-home ration (600 calories, 18-20 g of protein)
WaSH	Provision of water filters, hand washing stations, water storage bottles, soap and disinfectants and counselling	Counselling (of variable quality)
Psychosocial support	Screen for depressive symptoms using PHQ-9 questionnaire.<br>Counselling by study psychologist if PHQ score ≥10.<br>Refer to psychiatrist if PHQ-9 score ≥ 15 and/or suicidal ideation.<br><br>Promotion of positive thinking and problem-solving skills<br><br>Screen for substance abuse and exposure to second-hand smoke and alcohol use in husbands and counsel	Counselling (of variable quality)

Table A3. Comparison of Early Childhood interventions (including mother in postnatal period) between intervention and control group.

	Intervention Group	Control Group (Routine Care)
0-6 months: Mothers		
Health	Facilitate hospital visit at 6 weeks postpartum and encourage compliance to supplements.	Routine postnatal and early childhood care
Nutrition	Locally prepared snacks daily and milk 6 days a week (600 kcal, 20 g protein).<br><br>IFA (100 mg iron, 500 µg folic acid)<br><br>Calcium (1000 mg) and vitamin D (400 IU) supplementation daily<br><br>Multiple micronutrients daily	Food is provided through Anganwadis although compliance in the control group was not ensured and the uptake in general was low based on national level surveys.<br><br>Supplementary food for mothers, either cooked food or take-home ration (600 calories, 18-20 g of protein)<br><br>IFA for 180 days postpartum<br><br>Calcium and Vitamin-D3 for 180 days postpartum
WaSH	Continuation of all the WaSH interventions provided during pregnancy (water filters, water storage bottles, hand washing station, soap, and disinfectants)<br><br>Counselling on handwashing and hygiene practices (bathing the infant regularly, keeping the infant's surroundings clean, safe disposal of infant's feces, and handwashing before handling the baby).	Counselling of variable quality
Psychosocial support	Promotion of positive thinking and problem-solving skills<br>Screening mothers for depressive symptoms and management as required.<br><br>Counselling by study psychologist if PHQ score ≥10.<br>Referral to psychiatrist if PHQ-9 score ≥15 and/or suicidal ideation.	Counselling of variable quality
0-6 months: Infants		
Health	Educating mother and other family members to identify danger signs and in early care seeking for illness.<br>Facilitating referral to health facilities for infants with any danger signs or illness requiring facility-based management.<br>Counselling on timely immunization.	Routine postnatal and early childhood care

Nutrition	Initiation of breastfeeding (BF) within the first hour of birth.<br><br>Early lactation counselling for all mothers to prevent BF problems, resolution anytime during the first 6 months.<br><br>Counsel on exclusive BF till 6 months of age emphasizing exclusivity of BF from 3 up to 6 months of age.<br><br>Growth monitoring and management of IWG<br>Weight measurement on day 14 and thereafter monthly to identify IWG (<15th centile as per WHO Growth Velocity Standards, i.e. weight gain <20 g/day between ages day 14 to month 2; <15 g/d for months 3 and 4; <10 g/d for months 5 to 6) for all term infants.<br>Management through:<br>Lactation counselling,<br>Screening and treatment of infections,<br>Facility based management of IWG by senior pediatrician at Safdarjung Hospital after 15 days of continued efforts of lactation support and no medical cause is identified.<br>Additional support for LBW babies and preterm babies (even if not LBW).<br>BF support by lactation counselling through home visits in the first three months (biweekly in first month, weekly in second and third month, monthly from fourth to sixth month).<br><br>Offer expressed breastmilk feeding for preterm babies only after a breastfeed.<br><br>Extended support for preterm (through assessment of feeding, growth, and investigations within 4-6 weeks after birth) after discharge and ensuring that the advice given at the hospital is followed at home.<br><br>Support kangaroo mother care at home<br><br>Vitamin D 400 IU daily for all infants up to 6 months<br><br>Iron supplementation up to 6 months: VLBW from 2 weeks, LBW from 6 weeks	Promotion of optimal breastfeeding practices (early initiation of breastfeeding within one-hour)<br><br>Exclusive breastfeeding for the first six months, and continued breastfeeding for at least two years
WaSH	Continuation of all the WaSH interventions provided during pregnancy (water filters, water storage bottles, hand washing stations, soap, disinfectants)<br><br>Counselling on hygiene practices (safe preparation of food, storage and feeding of the child utilizing clean utensils and clean water for cooking and drinking).	Counselling of variable quality
Psychosocial support	Counselling, demonstration, and practice sessions for mothers at each home visit on early child play and responsive care. Identification of delayed development and timely referral for further management	Counselling of variable quality
6-24 months: Children		

Health	Educating mother and other family members to identify danger signs, and in early care seeking.<br><br>Facilitating medical management.<br><br>Counselling on feeding the child during and after illness.<br><br>Counselling on timely immunization<br><br>Provision of Albendazole (200 mg) for deworming starting 12 months of age and then 6 monthly	Routine early childhood care
Nutrition	Effective counselling on initiation of complementary feeding by preparing the mother and family 1-2 weeks prior to 6 months of infant's age.<br><br>Initiation of complementary feeding at 6 months of age and teaching the mother by demonstrations on how to prepare foods at home which can be fed easily to the child 6 months onwards.<br><br>Provision of daily food supplement with 125 kcal, 2.5 g protein up to 12 months 250 kcal energy and 5 g protein from 12 to 24 months that includes 80 to 100% RDA of micronutrients.<br><br>Counselling for intake of home-based food<br><br>Counselling and demonstration of responsive feeding to mother and family members<br><br>IFA (10 mg iron and 100 mcg folic acid) supplementation daily up to 24 months Lactation counselling for supporting continued BF till 24 months of age.<br><br>Growth monitoring (weight and length monthly)<br>Management of IWG (<25th centile as per WHO Growth Velocity Standards) by providing additional food supplements in form of snacks (125 kcal and 2.5 g protein up to 12 months, and 250 kcal energy and 5 g protein from 12 to 24 months) till Child has adequate weight gain for 2 consecutive months.<br>Nutritional counselling<br>Screening and treatment of infections<br>Home based management of moderate acute malnutrition through counselling, on preparation of augmented home-based foods.<br><br>Facilitating facility-based management of severe acute malnutrition.	Supplementary food (6 to 72 months of age): 500 calories, 12-15 g of protein.<br><br>For children with severe acute malnutrition, supplementary food increased to 800 calories, 20-25 g of protein.<br><br>IFA supplementation from 6 months to 24 months
WaSH	Continuation of all the WaSH interventions provided during pregnancy (water filters, water storage bottles, hand washing stations, soap, disinfectants)<br><br>Counselling on hygiene practices (safe preparation of food, storage and feeding of the child utilizing clean utensils and clean water for cooking and drinking).<br><br>Clean play area for children; provide play mats. Safe disposal of child's feces; provide potty.	Counselling of variable quality

Psychosocial support	Counselling on early child development.<br><br>Demonstration and practice session for mothers at each home visit on early child play and responsive care.<br><br>Identification of delayed development three-monthly or as response to parental concerns and timely referral to developmental psychologist.<br><br>Mothers<br><br>Promotion of positive thinking and problem-solving skills.<br><br>Screening mothers for depressive symptoms.<br>Counselling by study psychologist if PHQ score ≥ 10.<br>Referral to psychiatrist if PHQ-9 score ≥15 and/or suicidal ideation.	Counselling of variable quality

Table A4: Outcomes among women during preconception and pregnancy and among children at birth and 24 months of age which were used for estimating health benefits in monetary terms.

RTI/STI – reproductive tract infection/Sexually transmitted infections
BSID III - Bayley Scales of Infant and Toddler development, 3rd edition

Women	Children
Preconception<br>• Proportion RTI/STI<br>• Proportion anemic<br>• Proportion with depressive symptoms<br>• Proportion hypothyroid<br>Pregnancy<br>• Proportion with RTI/STI<br>• Proportion with Preeclampsia/eclampsia<br>• Proportion anemic<br>• Postpartum hemorrhage	Birth<br>• Proportion low birthweight<br>24-month of age<br>• Mortality including stillbirths.<br>• Proportion stunted at 24 months.<br>• Proportion wasted at 24 months.<br>• Mean cognitive and language score (BSID – III)

Table A5: The base, low- and best-case estimates used in the sensitivity analysis*.

Parameter	Base case	Low case	Best case
Costs (times unit cost)	1	1.3	0.7
Discount rate	3%	5%	1%
Growth rate of economy	5%	3%	7%
Value of Statistical Life (times Gross National Income)	100	51	160
Productive life span for VSLY	35	40	30

* The estimates for health outcomes used in sensitivity analysis were the point estimate (base case), lower bound of 98.3% CI (low case scenario) and upper bound of 98.3% CI (best case scenario) as shown in table

Note: Low case scenario sets the parameters at high levels for cost and low levels for benefits leading to lowest value for benefit cost ratio. Best case scenario sets the parameters at low levels for cost and high levels for benefits leading to lowest value for benefit cost ratio.

Table A6. Incremental monetary benefits, costs, benefit cost ratio and net monetary benefit of the integrated intervention package for different comparisons in USD PPP excluding mortality benefits.

Period	Outcome	Preconception vs Control	Pregnancy and Early Childhood vs Control	Preconception, Pregnancy and Early Childhood Vs Control
Preconception	RTI/STI	64.9	-29.7	59.2
	Anemia	307.7	-36.8	318.4
	Depression	-98.4	-127.5	24.2
	Hypothyroidism	0.9	-21.1	9.6
Pregnancy	RTI/STI	17.4	90.5	101.4
	Preeclampsia/Eclampsia	59.5	124.8	197.1
	Postpartum Hemorrhage	2.8	-10.5	36.0
	Anemia	121	396.6	241.2
Childhood	Low birthweight	1138.6	679.2	1116.7
	Stunting at 24 months	-130.9	2193	2321
	Wasting at 24 months	-276.5	502.9	488.4
	Cognition at 24 months	753.4	1339.4	2427.6
Benefits		1960.4	5100.8	7340.8
Cost		1441.1	2926.8	4367.9
BCR		1.4	1.7	1.7
Net Monetary Benefit		519.3	2174.0	2972.9

Table A7. Costs, benefits, and benefit cost ratio in USD PPP considering the factorial study design of WINGS.

Period	Outcome	Preconception (A + B) vs No Preconception (C + D)	Pregnancy and Early Childhood (A + C) vs No Pregnancy and Early Childhood (B + D)
Preconception	RTI/STI	77.8	-17.7
	Anemia	329.2	-16.3
	Depression	43.6	-6.5
	Hypothyroidism	15.2	-5.7
Pregnancy	RTI/STI	15.0	86.5
	Preeclampsia/Eclampsia	47.5	119.1
	Postpartum Hemorrhage	20.3	10.7
	Anemia	47.5	297.6
Childhood	Low birthweight	759.1	341.6
	Stunting at 24 months	-71.3	9839.4
	Wasting at 24 months	-200.7	2419.9
	Cognition at 24 months	-133.0	674.0
Benefits in USD PPP		1871.0	15249.5
Cost in USD PPP		1441.1	2926.8
BCR		1.3	5.2
Net Monetary Benefit		429.9	12,322.7

Note: The factorial design in WINGS enables us to compare groups that received interventions during different phases of the study with groups that did not receive the comparable interventions. Two such comparisons were possible: 1) groups receiving interventions during preconception vs groups not receiving interventions during preconception and 2) groups receiving pregnancy and early childhood interventions and groups which didn't receive pregnancy and early childhood interventions
